# A paradox: Fe^2+^-containing agents decreased ROS and apoptosis induced by CoNPs in vascular endothelial cells by inhibiting HIF-1α

**DOI:** 10.1042/BSR20203456

**Published:** 2021-01-08

**Authors:** Wenfeng Zhu, Yake Liu, Wei Wang, Zihua Zhou, Jin-hua Gu, Zexu Zhang, Huanjian Sun, Fan Liu

**Affiliations:** 1 Department of Orthopaedics, Affiliated Hospital of Nantong University, Nantong, Jiangsu Province, China; 2 Department of Orthopaedics, The Sixth Affiliated Hospital of Nantong University, Yancheng, Jiangsu Province, China; 3 Orthopaedic Laboratory, Affiliated Hospital of Nantong University, Nantong, Jiangsu Province, China; 4 Department of Clinical Pharmacy, Affiliated Maternity and Child Health Care Hospital of Nantong University, Nantong, Jiangsu Province, China

**Keywords:** CoNPs, Fe2+-containing agents, hypoxia-inducible factor, vascular endothelial cells

## Abstract

Cobalt nanoparticles (CoNPs) released from hip joint implants are known to have a toxic effect on several organs probably through increasing reactive oxygen species (ROS). Ferrous ion (Fe^2+^) is well-known to enhance oxidative stress by catalysing the production of ROS. However, in our pilot study, we found that Fe^2+^ conversely inhibited the ROS production induced by CoNPs. To elucidate the underlying mechanism, the present study treated vascular endothelial HUVEC and HMEC-1 cells with CoNPs alone or in combination with ferrous lactate [Fe(CH_3_CHOHCOO)_2_], ferrous succinate [Fe(CH_2_COO)_2_], and ferrous chloride (FeCl_2_). CoNP toxicity was evaluated by measuring cell viability, rate of apoptosis and lactose dehydrogenase (LDH) release, and intracellular ROS levels. Treatment with CoNPs decreased cell viability, LDH release, and ROS production and increased apoptosis. CoNPs increased hypoxia-inducible factor-1α (HIF-1α) protein level and mRNA levels of vascular endothelial growth factor (*VEGF*) and glucose transporter 1 (*GLUT1*) downstream of HIF-1α signalling. Silencing HIF-1α attenuated CoNP toxicity, as seen by recovery of cell viability, LDH release, and ROS levels and reduced apoptosis. CoNPs caused a pronounced reduction of Fe^2+^ in cells, but supplementation with Fe(CH_3_CHOHCOO)_2_, Fe(CH_2_COO)_2_, and FeCl_2_ restored Fe^2+^ levels and inhibited HIF-1α activation. Moreover, all three Fe^2+^-containing agents conferred protection from CoNPs; Fe(CH_3_CHOHCOO)_2_ and Fe(CH_2_COO)_2_ more effectively than FeCl_2_. In summary, the present study revealed that CoNPs exert their toxicity on human vascular endothelial cells by depleting intracellular Fe^2+^ level, which causes activation of HIF-1α signalling. Supplements of Fe^2+^, especially in the form of Fe(CH3CHOHCOO)_2_ and Fe(CH_2_COO)_2_, mitigated CoNP toxicity.

## Introduction

Cobalt-chromium (CoCr) alloy casting implants have been extensively used in total hip arthroplasties, especially in younger and more active patients. Compared with early metal-on-polyethylene bearing implants, CoCr metal-on-metal (MOM) resurfacing implants improve wear characteristics, thereby reducing wear-induced osteolysis, the main cause of loosening and dislocation of prostheses [1,2]. However, numerous studies have demonstrated that CoCr implants release a large amount of very small wear particles and metal ions [1,2]. Particles smaller than 50 nm in size have been reported. CoCr alloy is composed of 62% cobalt (Co) and 28% chromium (Cr). Cobalt nanoparticles (CoNPs) are the most common degradation products of MOM implants. Once the diameter of CoCr materials reaches the nanoscale, the nature of their biological effects changes [2]. Nano-sized Co particles can get incorporated into the periprosthetic tissue or enter the bloodstream and then reach organs such as heart, liver, kidney, and brain [1–4] and the toxicity of CoNPs has been observed in fibroblasts, U937 cells, peripheral blood mononuclear cells, and alveolar macrophages [5–7]. The deposition of CoNPs in tissues may trigger oxidative stress and inflammatory reactions, which could potentially lead to tissue necrosis and organ dysfunction [1–4].

Hypoxia-inducible factors (HIFs) are transcription factors that mainly mediate the cellular response to hypoxia. HIFs are heterodimers consisting of oxygen-sensitive α subunits (HIF-α) and a stable, constitutively expressed β subunit (HIF-β). Under normal cellular oxygen tension, HIF-1α subunits are hydroxylated on two prolines by an iron [Fe(II)]-dependent prolyl-hydroxylases (PHDs), which is subsequently degraded through the ubiquitin–proteasome pathway [8]. Decreased oxygen levels can inhibit PHD-dependent hydroxylation of HIF-1α subunits leading to the stabilisation of HIF-1α. Stabilised HIF-1α translocates to the nucleus and mediates a robust induction of a battery of target genes, which contain the hypoxic response element (HRE) [8]. A growing number of studies show that PHD activity is affected not only by oxygen tension but also by Fe(II) concentration. Introducing the iron chelator desferrioxamine or transition metals, such as Co, Mn, and Ni, also attenuates PHD activity, resulting in HIF-1α activation [9]. It is not completely understood why transition metals impair PHD activity. Possible reasons could be that transition metals interfere with the interaction between Fe(II) and PHD [9] or transition metal-induced reactive oxygen species (ROS) prevents the redox reaction of Fe(II) to Fe(III) [7]. Although the underlying mechanisms are unclear, it has been shown that the activation of the HIF pathway is closely linked to the toxicity mediated by CoNPs and Co^2+^ in three types of human macrophages [7].

Although HIFs help the cell adapt to hypoxia, HIFs are known to cause various adverse effects on the cell, such as oxidative stress, inflammatory response, apoptosis, and autophagy. Oxidative stress is associated with the overproduction of ROS and reactive nitrogen species (RNS). It has been found that HIFs promote ROS and RNS production by up-regulating the expression of NADPH oxidases (NOXs) and nitric oxide synthases (NOSs), by binding to the HREs in the promoter regions of *NOX* and *NOS*, respectively [10]. Knockdown of HIFs or mutation of HREs in *NOXs* and *NOSs* decreased the generation of ROS and RNS in cells under hypoxic conditions. HIFs also induced the expression of inflammatory factors (e.g., IL-1β, IL-6, and TNF-α) [11,12] and regulators of apoptosis, pyroptosis and autophagy, such as p53, Bcl-2, pyruvate dehydrogenase kinase 4, and BCL2/adenovirus E1B 19-kDa-interacting protein-3 [13–15].

Iron is crucial for cellular functions including respiration, oxygen transport, DNA synthesis, energy production and cell proliferation [16]. Iron deficiency causes many diseases, such as anemia and heart failure [16,17]. More than 20% of women experience iron deficiency, therefore immediate supplementation with iron from iron-rich foods and iron agents is necessary for the normal development of a fetus [16]. Ferrous ion (Fe^2+^) is also well known to enhance oxidative stress by catalysing the production of ROS especially in iron overload status. In our pilot study, we originally aimed to compare the ability of CoNPs and iron to induce the production of ROS. However, we carelessly added Fe^2+^-containing agents to cells exposing to CoNPs. Rather surprisingly, Fe^2+^-containing agents, instead of enhancing the toxicity of CoNPs, decreased ROS induced by CoNPs in turn.

The present study initially focused on the toxic effects of CoNPs on vascular endothelial cells because CoNPs derived from CoCr alloy implants can easily enter the bloodstream and get deposited on the vascular endothelium, due to their small size and high mass [4]. Moreover, the results from this study show that ferrous lactate [Fe(CH_3_CHOHCOO)_2_], ferrous succinate [Fe(CH_2_COO)_2_], and ferrous chloride (FeCl_2_) conferred protection to vascular endothelial cells from CoNPs, at an appropriate range of concentration, by inhibiting HIF-1α signalling. These results suggest a protective role for ferrous agents, after total hip arthroplasties using CoCr MOM implants.

## Materials and methods

### Cell culture

Two vascular endothelial cell lines, HUVEC and HMEC-1, were purchased from American Type Culture Collection (ATCC, Manassas, VA, U.S.A.). The cells were grown in DMEM (Gibco-Invitrogen, Grand Island, NY, U.S.A.) supplemented with 10% foetal bovine serum (FBS) (Invitrogen) and 1% antibiotic–antimycotic (Invitrogen). Cells were maintained in a humidified atmosphere consisting of 5% CO_2_ and 95% air at 37°C.

### Preparation of cell medium containing CoNPs

CoNPs were purchased from Sigma (Shanghai, China, particle size < 50 nm; purity ≥ 99%). CoNPs are cobalt metal with high purity, thus they are in zero-valent state and insoluble in cell culture medium. CoNPs pellets are mainly spherical. CoNPs were accurately weighed, sterilised at 180°C for 4 h, and then added to cell culture medium. The culture medium was sonicated for 15 min using ultrasonic oscillators (Ningbo Sklon Lab Instrument Co., Ltd; Shanghai, China) to completely disperse CoNPs in the culture medium. The CoNPs sample was reliable and effective until CoNPs were like fine sand uniformly deposited on the bottom of the sample well. To ensure reliable repeatability, three replicate wells were set for each concentration. Based on the results from our pilot experiments, concentrations of CoNPs ranging from 0–1000 μM were selected.

### Electron microscope observation

Cell pellets were fixed in 0.1 M phosphate buffer and 2% glutaraldehyde and embedded in epon resin with Epoxy Embedding Medium Kit (Sigma), following instructions from the kit provider. The sections were analysed with a Field Emission Gun-Environmental Scanning Electron Microscope (Quanta 200, FEI Company, Netherlands) in STEM mode.

### Treatment with ferrous agents

HUVEC and HMEC-1 cells were cultured in the cell medium containing CoNPs. Various concentrations of Fe(CH_3_CHOHCOO)_2_, Fe(CH_2_COO)_2_, and FeCl_2_ were added to the cells. Cells went through all the measurements 24 h after the treatments were done.

### Cell viability assay

The effect of CoNPs on the viability of vascular endothelial cells was assessed using the MTT assay. HUVEC and HMEC-1 cells were plated into 96-well culture plates (5 × 10^3^ cells per well) and then exposed to CoNPs. Culture medium served as the control in each experiment. Optical density was measured at 570 nm using a microplate reader.

### Lactose dehydrogenase release assay

A lactose dehydrogenase (LDH) assay kit (Beyotime Biotechnology, Shanghai, China) was used to test LDH release into culture medium. The method was according to the manufacturer’s protocol. The samples were assessed using a microplate reader.

### Apoptosis assessment using flow cytometry

An apoptosis detection kit provided by Beyotime Biotechnology was used to detect and quantify apoptosis in vascular endothelial cells. Briefly, cells were trypsinised and resuspended at a concentration of 1 × 10^6^/ml in diluted binding buffer and labelled with 10 μl of annexin V-FITC. Cells were incubated for 30 min at room temperature in dark, followed by a 5-min incubation with 5 μl of PI. Subsequently, 400 μl of 1× binding buffer was added to each tube. Flow cytometric analysis was performed to monitor the annexin V and DNA-bound PI. Data acquisition and analysis were performed using the FlowJo software.

### Hoechst33258 and PI staining

Cell apoptosis was also observed by a dual staining with Hoechst33258 (Beyotime Biotechnology) and PI. HUVECs were stained with 5 mg/l Hoechst 33258 (Sigma) for 30 min at 37°C, followed by a 5-min incubation with 5 μl of PI. The cells were visualised under a fluorescence microscope with standard excitation filters.

### Detection of intracellular ROS, lipid hydroperoxide and malondialdehyde

Intracellular ROS levels were evaluated using 2′,7′-dichlorofluorescin diacetate (DCFHDA) (Beyotime Biotechnology). DCFHDA can form the fluorescent compound dichlorofluorescein in the presence of ROS. DCFH-DA (10 μM) was added to cells. After the incubation for 20 min at 37°C, the cells were washed using MEM without serum, at least five times. Labelled cells were trypsinised, resuspended in PBS supplemented with 5% FBS, and analysed by flow cytometry (Fortessa, BD Biosciences). A minimum of 10000 cells were analysed per condition.

Lipid peroxidation causes the formation of the highly unstable and reactive intermediary product, LPO, and the end product, malondialdehyde (MDA). LPO level was measured using a Lipid Hydroperoxide Assay Kit (Caymen Chemical Co., Shanghai, China). LPO was initially extracted from the cell sample to avoid the interference of intracellular iron ions in the assay. LPO then reacted with a Fe^2+^ agent provided by the kit to yield Fe^3+^. Fe^3+^ further reacted with thiocyanate anion (SCN^−^) to produce a purple-coloured Fe(SCN)_5_^2−^ product that can be quantified spectrophotometrically at 500 nm. The level of LPO was expressed as nmol/g protein. For MDA measurement, cell sample were lysed and then heated in boiling water bath for 15 min, followed by thawing on ice, and the optical density of the solution was measured at 532 nm. The results were expressed as nmol MDA/mg protein.

### Iron assay

Fe^2+^ and total iron levels in cells were measured using an iron assay kit (ab83366, Abcam). Briefly, samples were homogenised in 5× volumes of iron assay buffer on ice and then the supernatant was collected. Iron buffer and iron reducer were added respectively for testing Fe^2+^ and total iron levels. Consequently, the iron probe was added to each sample before mixing and incubating for 60 min. Optical density was immediately measured at 593 nm on a colorimetric microplate reader.

### Short interfering RNA transfection for HIF-1α silencing

The vascular endothelial cells were seeded in six-well plates at a density of 5 × 10^6^ cells/well, 16 h before transfection. Cells were transfected with three pairs of HIF-1α short interfering RNAs (siRNAs) (50 nM, GenePharma, Shanghai, China), using Lipofectamine 2000 (Invitrogen). A scrambled siRNA was used as control. Six hours post-transfection, cells were cultured in fresh growth medium with or without treatment with CoNPs. Cells were then harvested for detection of HIF-1α mRNA and protein levels by real-time polymerase chain reaction (PCR) and Western blotting.

### Quantitative real-time PCR

mRNA was extracted from the HUVEC and HMEC-1 cells using the TRIzol-based method. The extracted mRNA (approximately 1 μg) was reverse-transcribed into the first-strand of cDNA using RevertAid First Strand cDNA Synthesis Kit (Thermo Fischer Scientific, Waltham, Massachusetts, U.S.A.). By using SYBR ExScript RT-PCR kit (TaKaRa, Dalian, China), the synthesised cDNA was used for quantitative real-time PCR on an ABI 7300 Real-Time PCR System (Applied Biosystems, Foster City, CA, U.S.A.). All gene expression data are normalised to glyceraldehyde 3-phosphate dehydrogenase (GAPDH) expression levels. The reaction conditions were as follows: initial 94°C for 30 s, followed by 35 cycles at 94°C for 5 s, 58°C for 15 s, 72°C for 10 s, and final extension at 72°C for 10 min. Specific primers are shown in Table 1.

**Table 1:** Specific primers used in PCR assays

Gene name	Primer orientation	Sequences	Tm (°C)	Amplicon size
*HIF-1α*	Forward	5′-GAACGTCGAAAAGAAAAGTCTCG-3′	60	124
	Reverse	5′-CCTTATCAAGATGCGAACTCACA-3′		
*VEGF*	Forward	5′-AGGGCAGAATCATCACGAAGT-3′	61	75
	Reverse	5′-AGGGTCTCGATTGGATGGCA-3′		
*GLUT1*	Forward	5′-GGCCAAGAGTGTGCTAAAGAA-3′	61	201
	Reverse	5′-ACAGCGTTGATGCCAGACAG-3′		
*NOX1*	Forward	5′-GCACACCTGTTTAACTTTGACTG-3′	61	129
	Reverse	5′-GGACTGGATGGGATTTAGCCA-3′		
*NOX2*	Forward	5′-ACCGGGTTTATGATATTCCACCT-3′	60	135
	Reverse	5′-GATTTCGACAGACTGGCAAGA-3′		
*NOX4*	Forward	5′-CAGATGTTGGGGCTAGGATTG-3′	61	96
	Reverse	5′-GAGTGTTCGGCACATGGGTA-3′		
*NOS3*	Forward	5′-TGATGGCGAAGCGAGTGAAG-3′	61	129
	Reverse	5′-ACTCATCCATACACAGGACCC-3′		
*GAPDH*	Forward	5′-GGAGCGAGATCCCTCCAAAAT-3′	60	197
	Reverse	5′-GGCTGTTGTCATACTTCTCATGG-3′		

### Western blot

Cells were lysed on ice for 30 min using a lysis buffer (Thermo Fischer Scientific). The supernatants of the lysates were denaturation and then separated on a 4–12% Bis-Tris gel (Invitrogen). Proteins in gel was next transferred to a nitrocellulose membrane, and probed with anti-HIF-1α and anti-GAPDH primary antibodies (Abcam) overnight at 4°C. Membrane-bound primary antibodies were detected using the appropriate secondary antibodies. Equal loading of protein was ensured by measuring GAPDH expression.

### Statistical analysis

All experiments were performed in triplicates in each cell line and the data are shown as mean and standard deviation, of three separate experiments. All statistical analyses were performed with GraphPad Prism 5 software (La Jolla, California, U.S.A.) using one-way analysis of variance followed by Dunnett’s test to evaluate significance relative to control.

## Results

### The toxic effect of CoNPs on vascular endothelial cells

**Figure 1:**
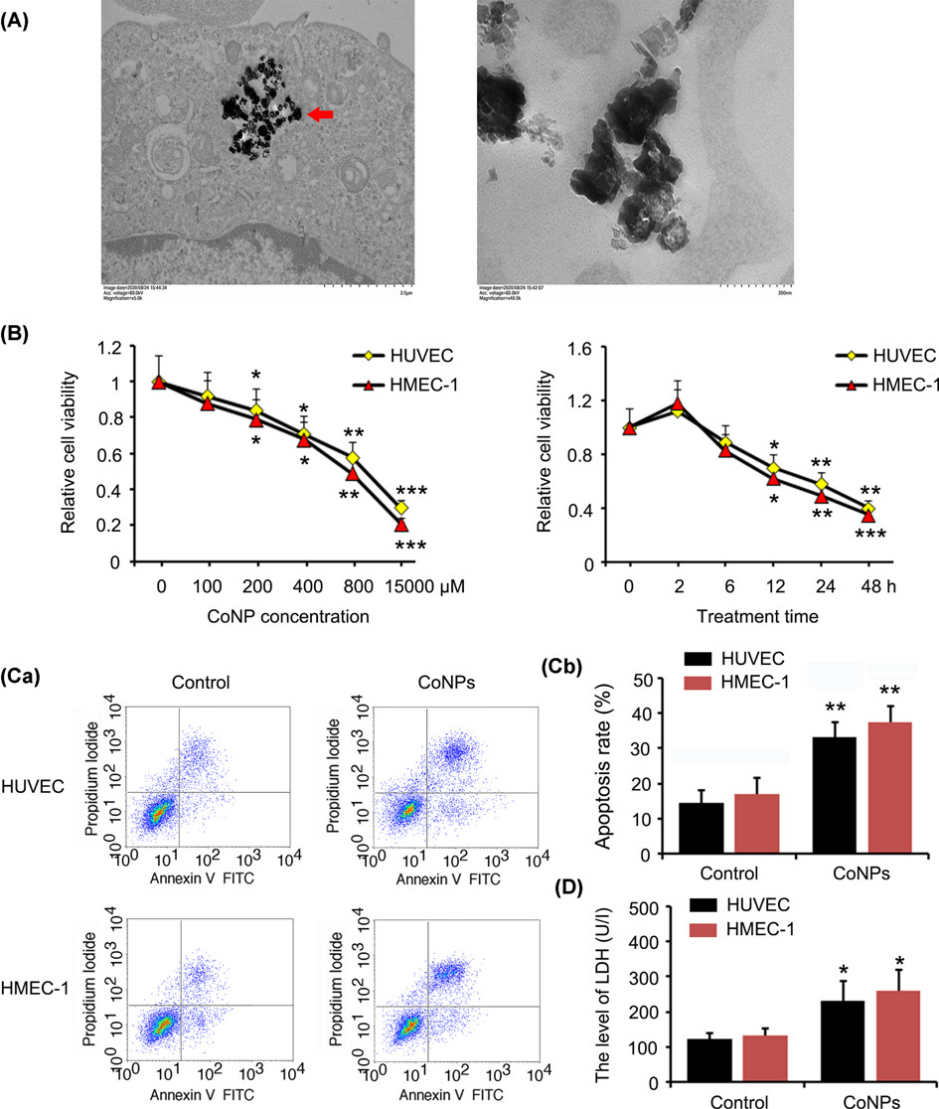
The toxic effect of CoNPs on vascular endothelial cells (**A**) Electron microscopic observation of HUVEC cells after treatment with CoNPs for 24 h. (**B**) HUVEC and HMEC-1 cells were exposed to varying concentrations of CoNPs (0, 100, 200, 400, 800, and 15000 μM) for 24 h or these cells were exposed to 800 μM CoNPs for 0, 2, 6, 12, 24, and 48 h. MTT was performed to evaluate cell viability. The viability of HUVEC and HMEC-1 cells were decreased by CoNPs in dose- and time-dependent manner. Apoptosis (**C**) and LDH assays (**D**) were performed, after HUVEC and HMEC-1 cells were treated with 800 μM CoNPs for 24 h. CoNPs increased the apoptosis and LDH concentration in culture medium. *P<0.05, **P<0.01 and ***P<0.001 vs. control group.

Using electron microscope, we observed that CoNPs entered into HUVEC cells after treatment with CoNPs for 24 h (Figure 1A). HUVEC and HMEC-1 cells were exposed to varying concentrations of CoNPs (0, 100, 200, 400, 800, and 15000 μM) for 24 h. MTT analysis showed that CoNPs decreased the viability of HUVEC and HMEC-1 cells in a dose-dependent manner (Figure 1B). Treatment with 800 μM CoNPs decreased the viability of HUVEC and HMEC-1 cells to approximately 50% (*P*<0.01). The present study also evaluated the toxic effect of 800 μM CoNPs at different time points (0, 2, 6, 12, 24, and 48 h). We found that 800 μM CoNPs marginally increased the viability of HUVEC and HMEC-1 cells after a very short incubation period (2 h). However, the viability HUVEC and HMEC-1 cells significantly decreased after incubation for 12 h with 800 μM CoNPs (*P*<0.05) and this decrease occurred in a time-dependent manner.

Treatment of HUVEC and HMEC-1 cells with 800 μM CoNPs for 24 h increased apoptosis (*P*<0.01, Figure 1C). The concentration of LDH in the culture medium was also found to be higher 24 h after treatment with 800 μM CoNPs (*P*<0.05, Figure 1D).

### CoNPs caused the activation of HIF-1α signalling and ROS production but decreased intracellular Fe^2+^ level

**Figure 2:**
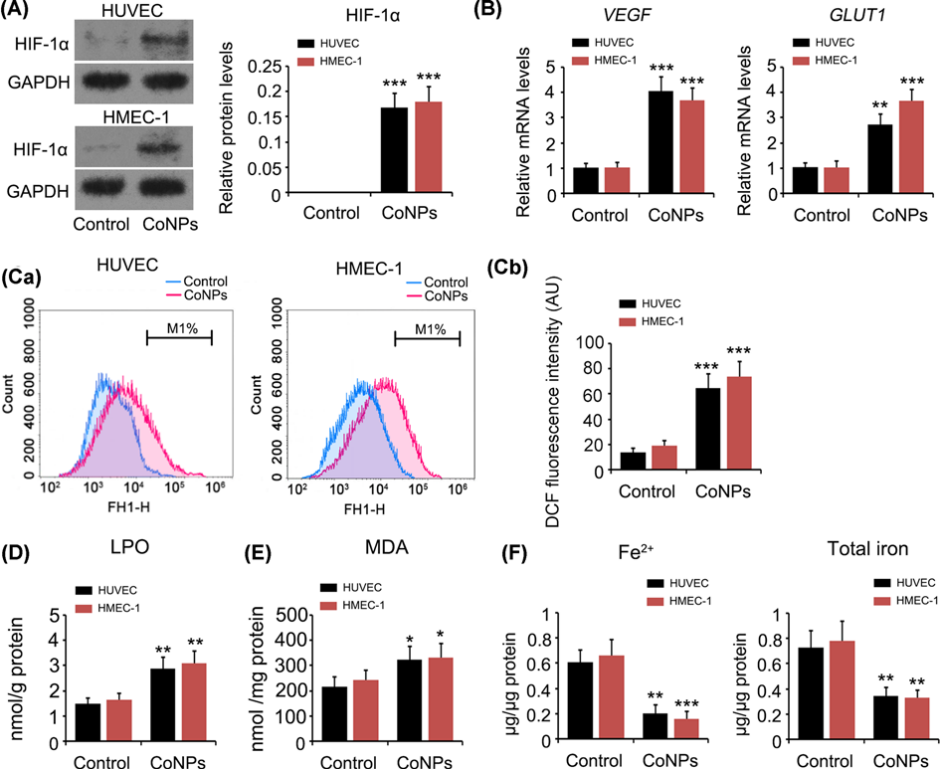
CoNPs caused the activation of HIF-1α signalling and ROS production but decreased intracellular Fe^2+^ level After HUVEC and HMEC-1 cells were treated with 800 μM CoNPs for 24 h, protein level of HIF-1α (**A**) and mRNA levels of VEGF and GLUT1 (**B**) were measured by Western blot and PCR assays, respectively. CoNPs increased HIF-1α, VEGF and GLUT1 in HUVEC and HMEC-1 cells. Intracellular ROS (**C**), LPO (**D**), MDA (**E**) as well as Fe^2+^ and total iron levels (**F**) were measured using flow cytometer or detection kits following the CoNPs treatment. CoNPs increased intracellular ROS, LPO and MDA, but decreased Fe^2+^ levels. *P<0.05, **P<0.01 and ***P<0.001 vs. control group.

To evaluate the effect of CoNPs on HIF-1α signalling, we measured the protein level of HIF-1α and mRNA levels of genes, *vascular endothelial growth factor* (*VEGF*) and *glucose transporter 1* (*GLUT1*), which are pre-transcriptionally regulated by HIF-1α. As seen in the Western blot, HIF-1α protein levels were higher in HUVEC and HMEC-1 cells under normoxia after treatment with 800 μM CoNPs for 24 h (*P*<0.001, Figure 2A). CoNPs also increased *VEGF* and *GLUT1* mRNA levels (*P*<0.01 or *P*<0.001, Figure 2B). Moreover, treatment with CoNPs dramatically increased ROS level in HUVEC and HMEC-1 cells (*P*<0.001, Figure 2C). LPO and MDA were increased by CoNPs as well (*P*<0.001, Figure 2D and *P*<0.01, Figure 2E, respectively). However, intracellular Fe^2+^ and total iron levels were found to be decreased after CoNPs treatment (*P*<0.01 or *P*<0.001, Figure 2F).

### Activation of HIF-1α signalling was associated with the toxic effect of CoNPs on vascular endothelial cells

**Figure 3:**
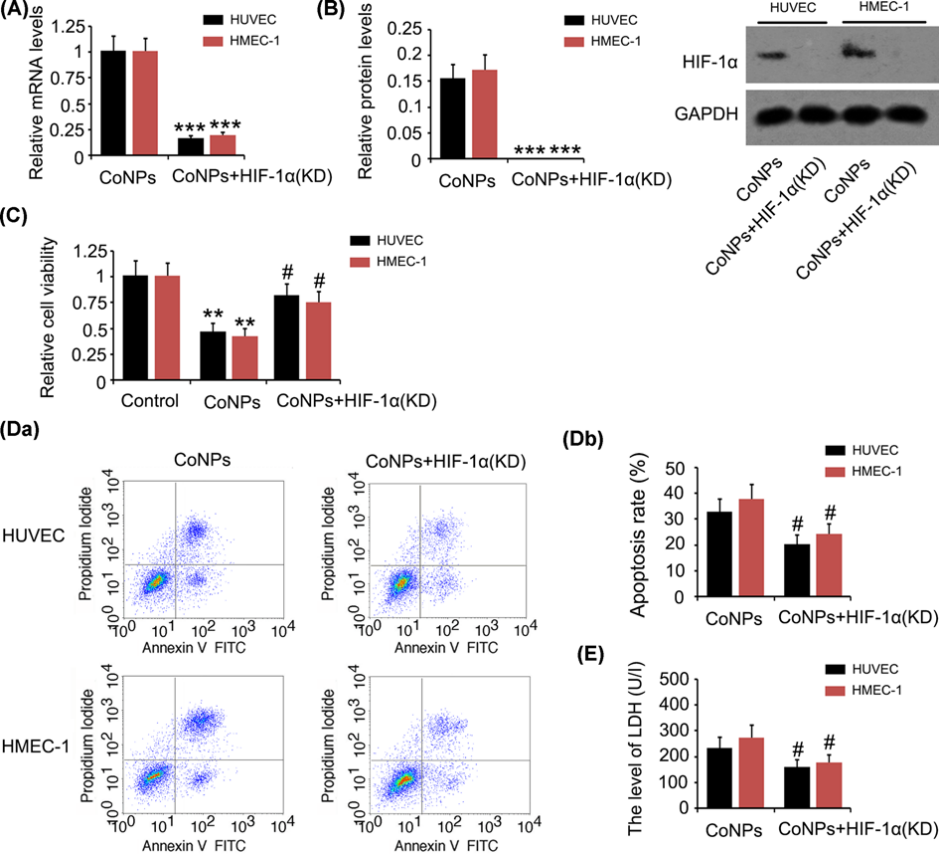
HIF-1α knockdown attenuated the toxic effect of CoNPs on vascular endothelial cells HIF-1α was knocked down before treating vascular endothelial cells with CoNPs. (**A**) PCR and (**B**) Western blot assays were performed to detect the mRNA level of HIF-1α in HUVEC and HMEC-1 cells. MTT (**C**), apoptosis (**D**) and LDH (**E**) assays were performed to evaluate the cell viability, apoptosis rate and damage in cell membrane, respectively. HIF-1α knockdown attenuated the toxic effect of CoNPs on vascular endothelial cells. **P<0.01 and ***P<0.001 vs. control group; ^#^*P*<0.05 vs. CoNPs group.

To determine whether HIF-1α activation mediates the toxic effect of CoNPs on vascular endothelial cells, we knocked down HIF-1α before treating vascular endothelial cells with CoNPs (*P*<0.001, Figure 3A,B). HIF-1α knockdown attenuated the reduction in cell viability caused by CoNPs (*P*<0.05 *vs.* CoNPs group, Figure 3C). The increase in apoptosis and release of LDH resulting from treatment with CoNPs were also suppressed with HIF-1α knockdown (*P*<0.05 *vs.* CoNPs group, Figure 3D,E).

**Figure 4:**
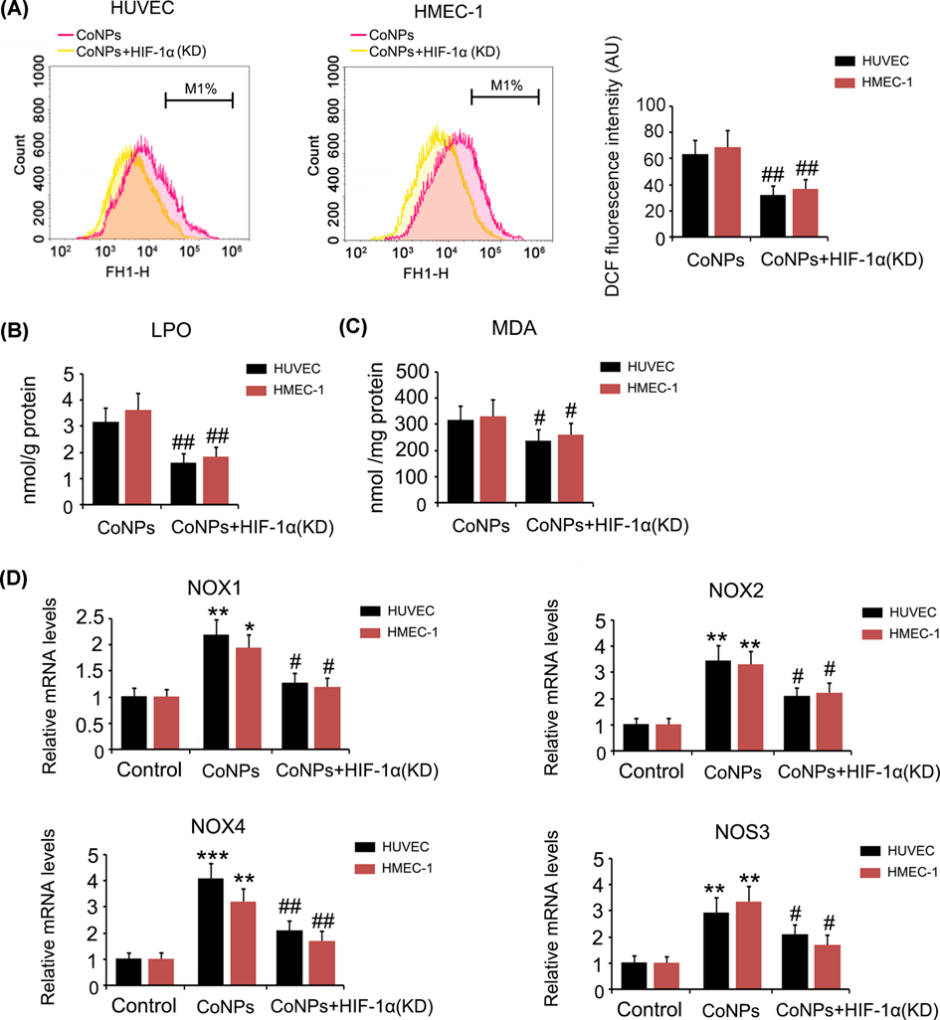
The regulatory effects of HIF-1α on intracellular ROS level in vascular endothelial cells treated with CoNPs Intracellular ROS (**A**), LPO (**B**) and MDA (**C**) were measured using flow cytometer or detection kits following the CoNPs treatment. Expression of NOX1, 2, and 4 as well as NOS3 were tested using PCR assay. Silencing HIF-1α blocked increase in ROS, LPO, and MDA in HUVEC and HMEC-1 cells induced by CoNPs. However, the reduction of Fe^2+^ level did not improve after HIF-1α knockdown. HIF-1α knockdown decreased expression of NOX1, 2, and 4 as well as NOS3 which were increased by CoNPs. *P<0.05, **P<0.01 and ***P<0.001 vs. control group; ^#^*P*<0.05 and ^##^*P*<0.01 vs. CoNPs group.

Silencing HIF-1α blocked increase in ROS in HUVEC and HMEC-1 cells induced by CoNPs (*P*<0.01, Figure 4A). The increase in LPO and MDA caused by CoNPs were also inhibited by HIF-1α knockdown (*P*<0.01, Figure 4B and *P*<0.05, Figure 4C, respectively). ROS-producing enzymes downstream of HIF-1α, including NOX1, NOX2, NOX4 and NOS3, were increased after CoNPs treatment (*P*<0.05, *P*<0.01 or *P*<0.001, Figure 4D), while the increase was suppressed by HIF-1α knockdown (*P*<0.05 or *P*<0.01 *vs.* CoNPs group). However, the reduction of Fe^2+^ and total iron level did not improve after HIF-1α knockdown (data not shown), suggesting that Fe^2+^ depletion mediated by CoNPs was not associated with HIF-1α signalling.

### Ferrous agents conferred protection against the toxicity of CoNPs in vascular endothelial cells

**Figure 5:**
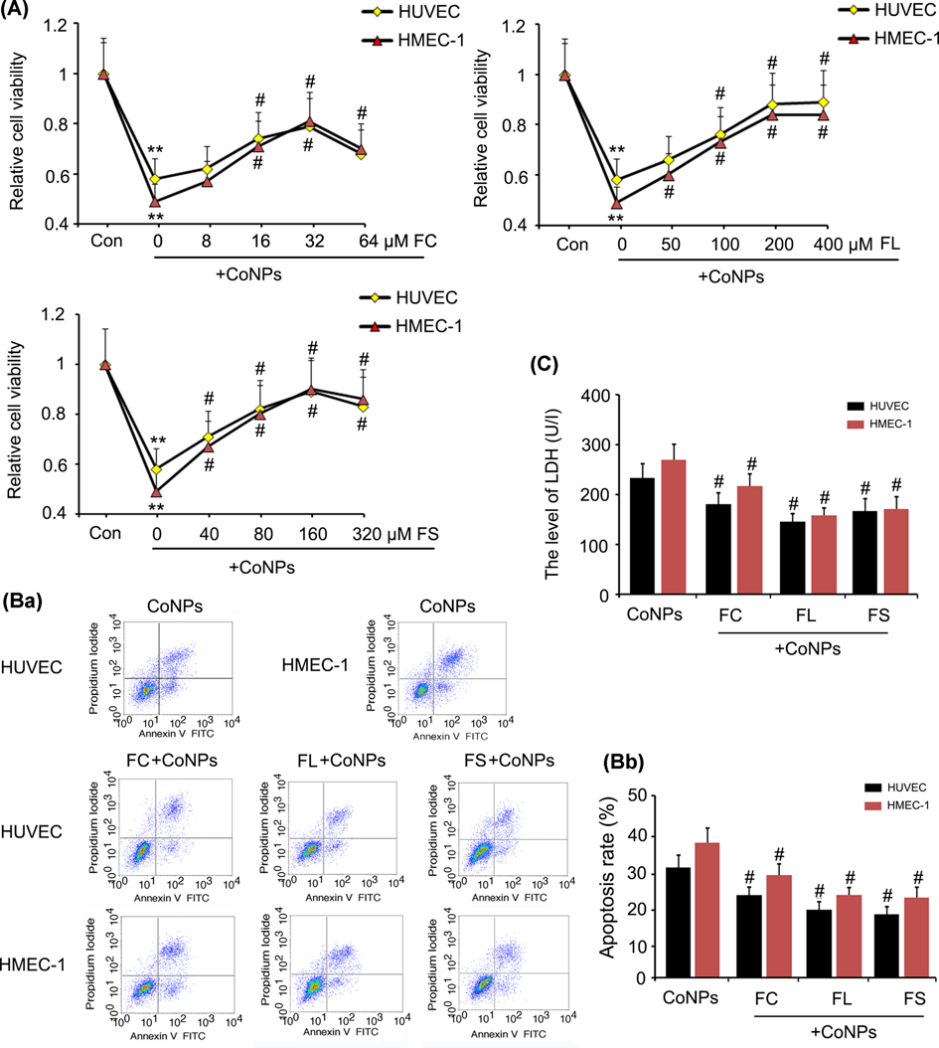
Ferrous agents conferred protection against the toxicity of CoNPs in vascular endothelial cells HUVEC and HMEC-1 cells were treated with 800 μM CoNPs in combination with Fe(CH3CHOHCOO)_2_, Fe(CH_2_COO)_2_, or FeCl_2_. MTT (**A**), apoptosis (**B**) and LDH (**C**) assays were performed 24 h after the treatments. All three ferrous agents improved cell viability and decreased the apoptosis and the LDH concentrations in culture medium. **P<0.01 vs. control group; ^#^*P*<0.05 vs. CoNPs group. Abbreviations: FC, ferrous chloride, (FeCl_2_); FL, ferrous lactate, [Fe(CH_3_CHOHCOO)_2_]; FS, ferrous succinate, [Fe(CH_2_COO)_2_].

CoNPs caused significant reduction of Fe^2+^ level in HUVEC and HMEC-1 cells, where Fe^2+^ plays important role in maintaining HIF-1α signalling in an inactive state. We added three ferrous agents, Fe(CH3CHOHCOO)_2_, Fe(CH_2_COO)_2_, and FeCl_2_ to vascular endothelial cells to compensate for the reduction in Fe^2+^ level caused by CoNPs, which was supposed to attenuated the toxic effects of CoNPs. All three ferrous agents improved HUVEC and HMEC-1 cell viability that was diminished by CoNPs (*P*<0.05, Figure 5A), while FeCl_2_ could not improve cell viability to the extent the other two ferrous agents could. All three ferrous agents decreased apoptosis in HUVEC and HMEC-1 cells and LDH concentrations in culture medium (*P*<0.05, Figure 5B,C), with more effective effects observed in Fe(CH_2_COO)_2_ and Fe(CH3CHOHCOO)_2_.

**Figure 6:**
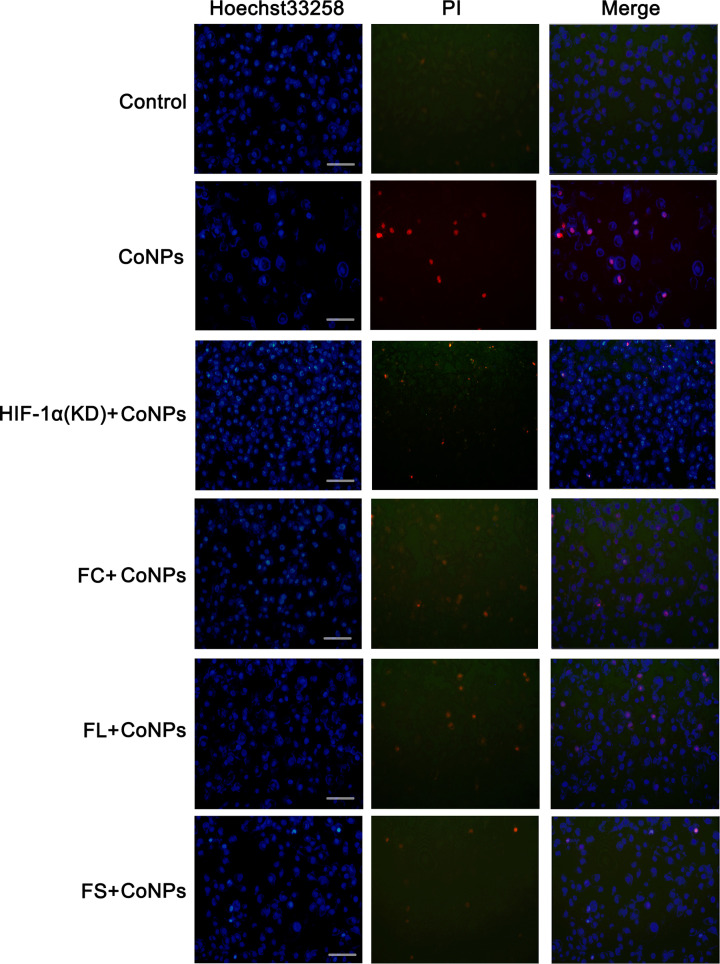
Hoechst33258 and PI staining The morphological changes of apoptosis were observed through a dual staining with Hoechst33258 and PI. The morphological changes of apoptosis include chromatin condensation (in early stage), and cell membrane fragmentation (in later stage). After Hoechst33258 staining, the nuclei of normal cells were normal blue, while the nuclei of apoptotic cells were dense stained or fragmented, and some of them turned white from blue colour. The nuclei of normal cells were not stained by PI, because PI can not pass through the complete cell membrane. However, the integrity of cell membrane was broken in the later stage of apoptosis, thus the nuclei of apoptotic cells can be stained. We observed that the cell number was reduced after treatment with CoNPs, with notable increase in PI staining. Silencing HIF-1α blocked the reduction in cell number caused by CoNPs, whereas a portion of cells showed chromatin condensation as indicated by Hoechst33258 staining. Treatments with Fe(CH3CHOHCOO)_2_, Fe(CH_2_COO)_2_, and FeCl_2_ attenuated the increase in PI staining induced by CoNPs.

The morphological changes of apoptosis were observed through a dual staining with Hoechst33258 and PI. The morphological changes of apoptosis included chromatin condensation (in early stage), and cell membrane fragmentation (in later stage). After Hoechst33258 staining, the nuclei of normal cells were normal blue, while the nuclei of apoptotic cells were dense stained or fragmented, and some of them turn white from blue colour. The nuclei of normal cells were not stained by PI, because PI can not pass through the complete cell membrane. However, the integrity of cell membrane was broken in the later stage of apoptosis, thus the nuclei of apoptotic cells can be stained. We observed that the cell number was reduced after treatment with CoNPs, with notable increase of PI staining (Figure 6). Silencing HIF-1α blocked the reduction in cell number caused by CoNPs, whereas a portion of cells showed chromatin condensation as indicated by Hoechst33258 staining. Treatments with Fe(CH3CHOHCOO)_2_, Fe(CH_2_COO)_2_, and FeCl_2_ attenuated the increase in PI staining induced by CoNPs.

**Figure 7:**
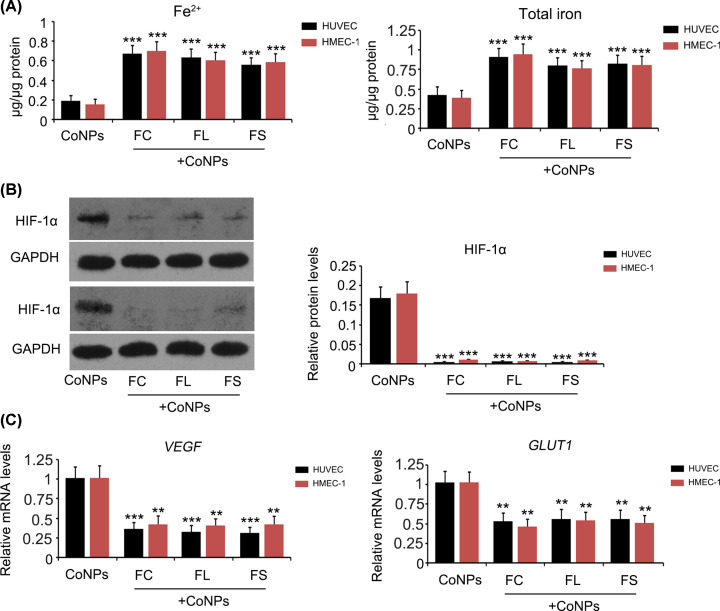
The regulatory effects of ferrous agents on HIF-1α signal and Fe^2+^ levels in vascular endothelial cells treated with CoNPs HUVEC and HMEC-1 cells were treated with 800 μM CoNPs in combination with Fe(CH3CHOHCOO)_2_, Fe(CH_2_COO)_2_, or FeCl_2_. Fe^2+^ and total iron levels were measured following the CoNPs treatment. (**A**) Expression of HIF-1α, VEGF and GLUT1 were tested using Western blot (**B**) or PCR assay (**C**). Treatment with these three agents increased Fe^2+^ and total iron levels in HUVEC and HMEC-1 cells that were exposed to CoNPs. All three ferrous agents inhibited increase in HIF-1α, VEGF, and GLUT1 induced by CoNPs. **P<0.01 and ***P<0.001 vs. CoNPs group. Abbreviations: FC, ferrous chloride, (FeCl_2_); FL, ferrous lactate, [Fe(CH_3_CHOHCOO)_2_]; FS, ferrous succinate, [Fe(CH_2_COO)_2_].

Treatment with these three ferrous agents increased Fe^2+^ and total iron levels in HUVEC and HMEC-1 cells that were exposed to CoNPs (*P*<0.001, Figure 7A). FeCl_2_ increased Fe^2+^ level in HUVEC and HMEC-1 cells more efficiently than Fe(CH_2_COO)_2_ and Fe(CH3CHOHCOO)_2_. Treatment with all three ferrous agents inhibited increase in HIF-1α protein (*P*<0.001, Figure 7B) and *VEGF* and *GLUT1* mRNA levels (*P*<0.01 or *P*<0.001, Figure 7C).

**Figure 8:**
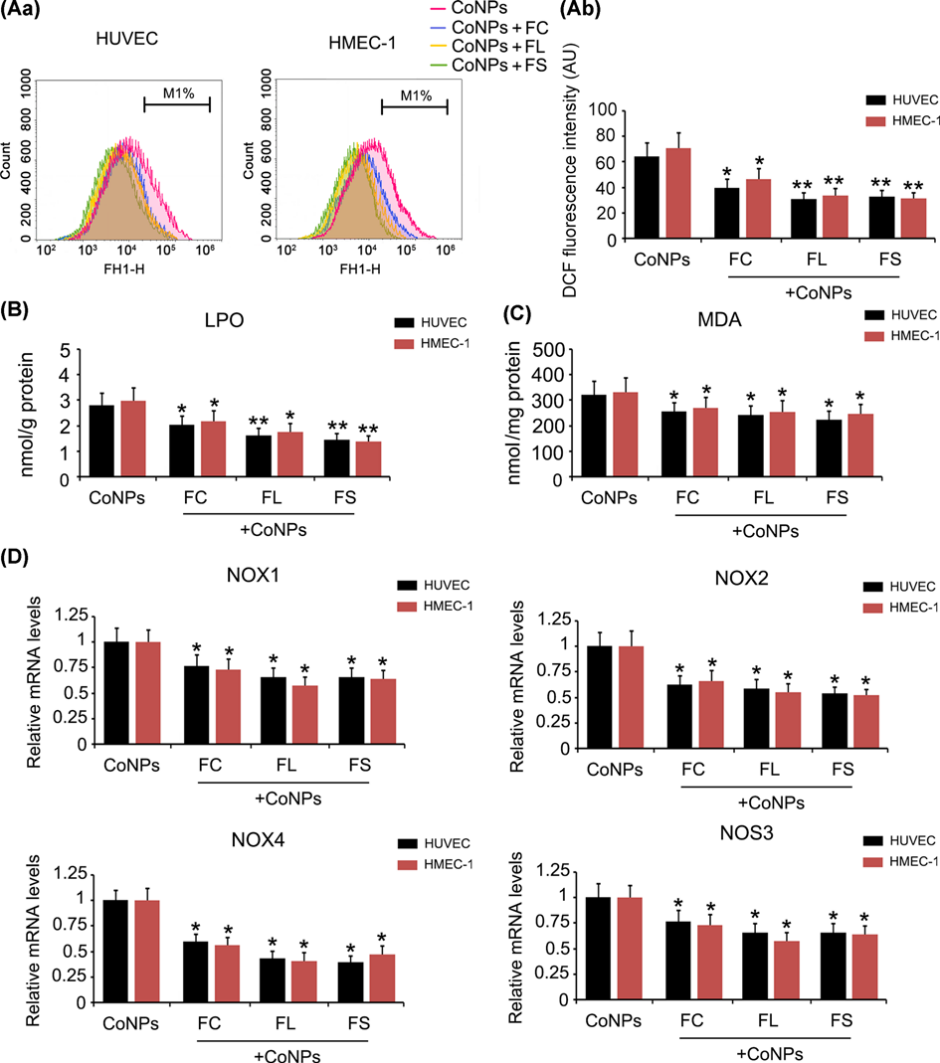
The regulatory effects of ferrous agents on intracellular ROS level in vascular endothelial cells treated with CoNPs HUVEC and HMEC-1 cells were treated with 800 μM CoNPs in combination with Fe(CH3CHOHCOO)_2_, Fe(CH_2_COO)_2_, or FeCl_2_. Intracellular ROS (**A**), LPO (**B**) and MDA (**C**) were measured using flow cytometer or detection kits following the CoNPs treatment. Expression of NOX1, 2, and 4 as well as NOS3 were tested using PCR assay (**D**). All three ferrous agents inhibited increase in ROS, LPO, and MDA in HUVEC and HMEC-1 cells induced by CoNPs. Moreover, all three ferrous agents decreased expression of NOX1, 2, and 4 as well as NOS3 which were increased by CoNPs. *P<0.05, and **P<0.01 vs. CoNPs group. Abbreviations: FC, ferrous chloride, (FeCl_2_); FL, ferrous lactate, [Fe(CH_3_CHOHCOO)_2_]; FS, ferrous succinate, [Fe(CH_2_COO)_2_].

Moreover, intracellular ROS, LPO and MDA levels, which increased upon treatment with CoNPs, were reduced in the presence of all three ferrous agents (*P*<0.05 or *P*<0.01, Figure 8). All three ferrous agents decreased CoNPs-mediated increase in NOX1, NOX2, NOX4 and NOS3 expression.

## Discussion

The toxic effects of CoNPs have earlier been reported in liver, kidney, and brain but few studies have investigated the influence of CoNPs on blood vessels [1–4]. After CoNPs enter the bloodstream, vascular endothelial cells are directly exposed to CoNPs. We found that CoNPs exerted their toxic effects on the vascular endothelium. Since the rate of apoptosis and LDH release also increased, along with inhibition of cell viability, CoNPs probably caused both apoptosis and necrosis of vascular endothelial cells. Most studies attribute the toxic effects of CoNPs to ROS production. However, not all antioxidants have been effective in attenuating toxicity. For example, l-ascorbic acid and α-tocopherol have been found to attenuate apoptosis, induced by CoNPs, by suppressing ROS production [18,19]. In contrast, glutathione failed to enhance cell viability of human macrophages after CoNPs treatment, although it decreased CoNP-induced ROS production [7]. These data suggest that the toxicity of CoNPs cannot be attributed to the adverse effects of ROS alone. Interestingly, we found that vascular endothelial cells had relatively strong resistance to CoNPs compared other types of cells (e.g. lymphocytes) [7]. One possible reason is that endothelial cells have a property that they closely connect to each us due to the tight junction. We guess that the tight junction between endothelial cells prevent the contact of CoNPS to the side and bottom of endothelial cells, only exposing the top of cells to CoNPS. Previous study showed lymphocytes are vulnerable to CoNPs. Lymphocytes are independently floating in the culture medium [7]. Therefore, CoNPS can contact to every sides of lymphocytes.

A few studies have found abnormal activation of HIF-1α signalling by CoNPs, even under normoxia. The primary function of HIF-1α signalling is to enable the cell to adapt to hypoxia, for example by increasing GLUT expression to elevate the efficiency of glucose transport and by inducing autophagy to derive energy from the degradation of the structural components of the cell [20]. However, activation of HIF-1α under normoxia is associated with many adverse effects. For instance, high glucose levels can trigger HIF-1α activation in H9c2 cardiomyoblasts under normoxia. Silencing HIF-1α attenuated apoptosis and inflammation in H9c2 cells under high glucose stress [21]. In addition, various reports indicate that HIF-1α induces the expression of NOXs and NOSs, which are key enzymes in cellular ROS production [10]. We have confirmed that HIF-1α activation is closely linked with the toxicity of CoNPs in vascular endothelial cells, because HIF-1α depletion attenuated apoptosis, LDH release, and ROS production caused by CoNPs. This is in agreement with the results from a study done by Nyga et al., who found that HIF-1α activation mediated the toxic effects of CoNPs in human macrophages [7].

HIF-1α activity is tightly controlled by Fe(II)-dependent PHD that induces the rapid degradation of HIF-1α under normoxia. However, we found that Fe^2+^ levels decreased in HUVEC and HMEC-1 cells after being treated with CoNPs. Although HIF-1α is involved in the regulation of intracellular iron levels, HIF-1α knockdown did not result in the recovery of Fe^2+^ levels, suggesting that reduction of Fe^2+^ level by CoNPs is not related to HIF-1α activation. Conversely, supplementation with Fe^2+^-containing agents to restore Fe^2+^ level in cells resulted in the degradation of HIF-1α and down-regulation of genes downstream of HIF-1α. These data suggest that the activation of HIF-1α induced by CoNPs is associated with the reduction of Fe^2+^ levels. The mechanisms by which CoNPs caused a reduction of Fe^2+^ level in cells, are not fully understood. According to the literature, CoNPs-induced ROS depletes large amounts of vitamin C in cells required for the oxidation of Fe(II) from Fe(III) [7]. Another possible reason is that Co^2+^ from CoNPs competes with Fe^2+^ for divalent metal-ion transporter-1 binding [22]. Divalent metal-ion transporter-1 is important for transporting iron ions but can also transport other divalent metal ions such as, Co^2+^ and Mn^2+^ [22]. However, both these reasons cannot fully explain all the results from CoNPs treatment. The present study helps confirm that ROS production induced by CoNPs is closely linked with HIF-1α activation, and HIF-1α activation is associated with Fe^2+^ reduction caused by CoNPs. Thus, ROS production is supposed to occur after Fe^2+^ reduction. Of course, existence of other mechanisms underlying CoNPs-induced ROS production before Fe^2+^ is reduced, cannot be ruled out. Further, the present study confirms that CoNP toxicity cannot be attributed to induce ROS alone; its toxicity is also associated with the induction of iron deficiency. Therefore, further work is needed to elucidate the mechanism behind CoNPs-induced reduction of Fe^2+^ level in cells.

In the present study, we have reported for the first time, that supplementing media with Fe^2+^-containing agents protected vascular endothelial cells from CoNPs toxicity. This finding might overthrow following traditional concept: CoNPs exert their toxic effects by triggering ROS overproduction; it is well-known that Fe^2+^ strengthens the oxidative capacity of ROS by a Fenton-type reaction (O_2_^●−^ + H_2_O_2_ → O_2_^+^ OH^−^ + OH). Thus, it is necessary to decrease Fe^2+^ levels to suppress the oxidative capacity of ROS. However, according to our data, CoNPs caused a notable reduction in Fe^2+^ levels, which causes HIF-1α activation and subsequently, ROS production. Therefore, supplementation with Fe^2+^-containing agents, within a proper range of concentrations, inhibited ROS induced stress and protected from CoNPs toxicity.

The present study also revealed that the protective effects of Fe(CH3CHOHCOO)_2_ and Fe(CH_2_COO)_2_ were more pronounced than those of FeCl_2_, although the efficiency of FeCl_2_ in enhancing intracellular iron levels was higher than the other two. After being released from Fe(CH3CHOHCOO)_2_ and Fe(CH_2_COO)_2_, Fe^2+^ forms weak acids with H^+^, whereas FeCl_2_ probably forms a strong acid. We found that the dosage range of FeCl_2_ for protecting cells was very narrow. Using high doses of FeCl_2_ would leave many Cl^−^ in the culture medium, thereby destroying the acid–base balance in the extracellular environment. In addition, CH3CHOHCOO^−^ and CH_2_COO^−^ might chelate Co^2+^ like they chelate Fe^2+^. This reaction might also attenuate CoNPs toxicity, but proving this hypothesis requires further investigation. Thus, the present study recommends the use of Fe(CH3CHOHCOO)_2_ and Fe(CH_2_COO)_2_ to protect vascular endothelial cells from CoNPs toxicity.

In summary, the present study revealed that HIF-1α activation mediated the toxic effects of CoNPs in human vascular endothelial cells. The activation of HIF-1α is associated with the reduction of Fe^2+^ level in cells, which is caused by CoNPs. Supplementation with Fe^2+^-containing agents, especially Fe(CH3CHOHCOO)_2_ and Fe(CH_2_COO)_2_ mitigated the toxic effects of CoNPs.

## Data Availability

The data used to support the findings of the present study are available from the corresponding author (Fan Liu) upon request.

## Abbreviations

CoCr: cobalt-chromium
CoNP: cobalt nanoparticle
DCFDA: 2′,7′-dichlorofluorescin diacetate
FBS: foetal bovine serum
Fe^2+^: ferrous ion
FeCl_2_: ferrous chloride
Fe(CH_3_CHOHCOO)_2_: ferrous lactate
Fe(CH_2_COO)_2_: ferrous succinate
GAPDH: glyceraldehyde 3-phosphate dehydrogenase
GLUT1: glucose transporter 1
HIF-1α: hypoxia-inducible factor-1α
HRE: hypoxic response element
LDH: lactose dehydrogenase
LPO: lipid hydroperoxide
MDA: malondialdehyde
MOM: metal-on-metal
NOS: nitric oxide synthase
NOX: NADPH oxidase
PCR: polymerase chain reaction
PHD: prolyl-hydroxylase
RNS: reactive nitrogen species
ROS: reactive oxygen species
siRNA: short interfering RNA
VEGF: vascular endothelial growth factor
